# Migrant mothers’ experiences of Caesarean section: a transcultural qualitative study

**DOI:** 10.3389/fpsyt.2023.1152810

**Published:** 2023-04-25

**Authors:** Juliette Rodriguez, Marie Rose Moro, Rahmeth Radjack

**Affiliations:** ^1^AP-HP, Department of Adolescent Psychiatry, Maison de Solenn, Cochin Hospital, Paris, France; ^2^Laboratoire de Psychologie Clinique, Psychopathologie, Psychanalyse, Université Paris Cité, Boulogne-Billancourt, France; ^3^Université Paris-Saclay, UVSQ, Inserm, CESP, Team DevPsy, Villejuif, France

**Keywords:** Caesarean section (C.S), emigrants and immigrants, migrant women, qualitative research and analysis, transcultural studies, obstetrics, maternity care

## Abstract

**Objectives:**

Migrant women face an increased risk of adverse obstetrical outcome and Caesarean section. The psychological experience of Caesarean section combines physiological, social, and cultural dimensions. This qualitative study explores the subjective experiences of first-generation migrant women who gave birth by Caesarean section.

**Methods:**

Seven qualitative, semi-directed interviews were conducted from January to March 2022 in a Paris maternity hospital, with women in their postpartum period who had had a scheduled or emergency Caesarean section and straightforward obstetrical outcomes. The presence of an interpreter-mediator was systematically offered. Thematic analysis of the interviews was carried out following the Interpretative Phenomenological Analysis (IPA) methodology.

**Results:**

Four themes were identified in the thematic analysis relating to the women’s experiences of Caesarean section: (1) The shock of the intervention combines disappointment, fear and early separation from the baby, (2) Pregnancy and delivery far from one’s family aggravates the psychological suffering caused by isolation and loneliness related to migration, (3) The lack of cultural representations of Caesarean section leads to negative preconceptions and hinders mental preparation, in contrast with traditional or medicalised childbirth, and (4) The women’s experiences of the medical follow-up highlights the importance of the continuity of care.

**Discussion and conclusion:**

Caesarean section, which is a physical break, re-enacts the symbolic break (cultural, social, familial) that follows on from emigration. Improvements in care include the need for a better preparation for Caesarean section, active efforts for care continuity, and the development of early prevention interviews and groups in maternity units.

## Background and introduction

Migrant women have a greater likelihood of risk-prone pregnancies, perinatal complications, premature childbirth, or Caesarean section (1–4). Higher rates of Caesarean section are observed among migrant women in large-scale studies. One Norwegian study in 2000, in an analysis of 17,000 births to non-European migrant women, found a prevalence of Caesarean section reaching 25%, while the average percentage for Norwegian women is 12%. Disparities according to cultural and geographical origin are considerable, with particularly high rates for women from the Philippines (25.8%) Brazil and Chile (24.3%) India and Sri Lanka (21.3%) and countries in the Horn of Africa (Somalia, Eritrea, Ethiopia) (20.5%) (5). A meta-analysis in 2013 found similar results from a synthesis of studies performed in 18 Western countries (Europe and North America). Among the 70 studies included, 69% evidenced different rates of Caesarean section between migrants and non-migrants. In particular, the rates are regularly higher among women from Sub-Saharan Africa, Somalia, and Southern Asia. In addition to this, the rates for emergency Caesarean section are higher among North African women, women from Western Asia, and Latin America. In the Paris region, a study by Linard using data from 2000–2002 found a proportion of 17% for Caesarean section among French women, versus 31% for women from Sub-Saharan Africa (6).

The factors suggested to explain this excess risk among migrant women are numerous. They include language and communication barriers, low socio-economic status, frail health of the mothers, high BMI, disproportion between uterus and foetus, previous uterine scarring, inadequate prenatal care, representations of pregnancy follow-up different from those of the country of origin, and isolation (1, 6–10). The precarious situations associated with migration are a considerable perinatal risk factor, as is isolation and absence of marital support, administrative status, and the existence or otherwise of health cover (3, 10). Command of the French language also appears a determining factor in the instatement of adequate pregnancy follow-up. Women who can neither read nor write French have a risk multiplied by three of not having the recommended pregnancy follow-up (10). For migrant mothers, fears associated with Caesarean section, often life-threatening in the country of origin, can combine with the experience of a cultural, religious or sexual transgression, and thus further compound the difficulties encountered for these parents starting a family far from their cultural landmarks and the support of their families (11). Anthropological studies on the perinatal period and obstetrics in traditional rural areas report negative cultural representations of Caesarean section (12–20). Women from Sub-Saharan Africa, West Africa or the Maghreb who have a Caesarean section run the risk of rejection by their husbands, or being branded as sterile, and are liable to be excluded from the social group if they can no longer bear children, or if they are held responsible for malformations in the baby (15, 16, 21, 22). Refusal to consent to Caesarean section is a frequent source of difficulties for caregivers in obstetric units, who argue compliance with medical decisions and the absolute need to save the lives of the mother and child, in the face of people who prefer to put their trust in divine will and who refuse the mutilation of Caesarean section (23). The intervention is most often dreaded or refused, since it signals to the community the woman’s inability to give birth (12, 13).

### Objectives of the study

This qualitative study aimed to explore the experiences of migrant women who have had a child by Caesarean section, so as to suggest lines for improvement in terms of psychological prevention and care provision for these vulnerable populations. The issue is approached from a transcultural and complementarist (24) point of view, so as to facilitate access to participants’ inner experiences, while at the same time taking their anthropological and cultural belonging into account. This requirement has taken the form of an integration into the design of our interview guide of probe questions enabling women to express their cultural perceptions, and also of the availability of interpreters in the research interviews to enable participants to use their native language. The main aim of the research was to explore the subjective experience of women from diverse cultural origins who had given birth by Caesarean section. The secondary objective was to propose lines for improvement in terms of psychological prevention and mental care for this vulnerable population.

## Materials and methods

### Ethics approval

The research protocol was reviewed and approved by the Paris-Cité University ethics committee in 2021 (ethics committee registration number n° IRB: 00012021–109). All participants were adults and provided written informed consent to the study.

### Methods

This was a single-centre, exploratory, qualitative study. The participants were recruited in the Port-Royal maternity unit, Cochin Hospital (APHP) in Paris during their post-natal stay. They had no previous links with the research team. Inclusions took place from January to March 2022 in the following manner: after an interview with the team of midwives and ascertainment of their eligibility, one researcher (JR) visited the rooms of the patients who were eligible to propose a face-to-face interview. The information letter and the consent form were given to the patients, who were allowed a period of reflection before agreeing to participate (see Annexe 1). Then an appointment was made, usually for the day after inclusion, according to patient preference and the availability of the ISM (Inter Services Migrants) interpreters. The participants could be accompanied by a person of their choice for the interview. Recruitment continued until theoretical saturation of the data, i.e. when the inclusion of new participants reaches a point of redundancy and all perspectives appear to have been explored (25, 26).

The inclusion criteria were as follows:

- Being a first-generation migrant mother, however long she has been in France
- Having recently given birth by Caesarean section, with straightforward sequellae for mother and child
- Caesarean section performed in emergency or planned.

The exclusion criteria were as follows:

- Mothers with a decompensated acute psychiatric disorder
- Mothers of new-born infants with serious neo-natal complications
- Mothers with serious obstetrical complications.

No financial remuneration was offered for participation in this research. The collection of informed consent and the perusal of the information letter were repeated before the interview and the data collection, with the help of the interpreter. An interpreter-mediator was systematically called on for the research interviews if the participant’s mother tongue was not French, so as to facilitate access to the cultural concepts and representations of the participant, or to detail complex emotions is their own language. The participants’ viewpoints were collected using a semi-structured interview guide specifically designed for this research. The interview guide included a limited number of open probe questions enabling the conversation to start on the research themes, while at the same time attempting to foster an interview that was as fluid and spontaneous as possible (Table 1). The duration of the interview was adapted to the state of fatigue of the participants, who were informed of the possibility of discontinuing the interview at any time. In case of manifestations of mental suffering in the course of the interviews, participants were asked if they consented to the transmission of this information to the health-care team in the maternity follow-up unit with a view to instatement of psychological care.

**Table 1:** Interview guide.

1. You recently gave birth. How are you feeling today?
2. How did your pregnancy go? Can you tell me how things went and who accompanied and assisted you, medically or non-medically?
3. How did your close and broader family accompany you in this period of pregnancy and childbirth??
4. There are many ways to bring a child into the world. In your family, how is childbirth viewed? What were your own thoughts and expectations for your delivery?
5. Can you tell me about the delivery of your child? How was the decision to perform Caesarean section reached?
6. Tell me about your first encounter with your child. How did the first few days go?
7. What accompaniment did you have by professionals in the maternity ward following the Caesarean section?
8. Do you have anything to add about your experience of Caesarean section?

### Data analysis

The interviews were recorded on a secure recorder in anonymous form, and were transcribed word-for-word. The analysis was performed using Nvivo (QSR International) qualitative analysis software. The analysis implemented Interpretative Phenomenological Analysis (IPA), which aims to provide an in-depth exploration of the ways in which subjects experience and give meaning to a phenomenon, from the study of their narratives on the subject (27, 28). The original interviews were coded line by line in full, using notations that were as close as possible to the original. Each unit of meaning (sentence, paragraph, verbatim) was translated into primary notations, and these were then grouped in codes, which in turn were grouped in themes and meta-themes. The thematic analysis grid was derived from the categories developed in inductive manner from the themes obtained. The analysis of the interviews was conducted by three researchers (JR, RR, MRM) (triangulation to ensure concordance between the analyses and the results).

## Results

1. Description of the sample

We interviewed seven women of various cultural origins: Australia, Spain, the Gambia (Soninke), Senegal (Wolof), Ivory Coast, Cameroon and Mali (Bambara). They had been in France for durations of 1 month to 5 years. The interviews were conducted from day 2 to day 4 after childbirth. Four of the Caesarean deliveries were emergency interventions, among which two followed on from a failed attempt to trigger labour. Two interviews were conducted without an interpreter, as none was available and the women were from African countries where French is commonly used. In line with our protocol, we interviewed only first-time migrant women.

Four of the seven participants were living with their partner and had their partner present during their stay in the maternity hospital. For those whose husbands were not present during the interview, this was due to situational factors (having to look after the eldest child, or the partner having gone to make the administrative declaration of birth) and not to a refusal on the part of the mother or to a lesser paternal investment.

As in any qualitative study, the small number of participants does not allow statistical conclusions to be drawn from the descriptive table of participants.

The interviews were conducted from January to March 2022 (Table 2). The duration of the interviews was 20 min to 1 hour, with an average of 53 min.

**Table 2:** Participant characteristics.

	Native language /country of origin	Marital status	Arrive in France	Parity	Reason for Caesarean	Psychological follow-up for:	People present during the research interview
M01	English (Australia)	With a partner	2 months	G1P1- 1st Caesarean	Caesarean during labour	–	Partner
M02	Spanish (Spain)	With a partner	Several years	G1P1- 1st Caesarean	Caesarean planned before labour	–	Partner
M03	Soninké/ Spanish (Gambia/Spain)	With a partner	2 and a half years	G1P1- 1st Caesarean	Caesarean during labour	Depression	Alone
M04	Wolof (Senegal)	With a partner	5 years	G2P2- 2nd Caesarean	Emergency Caesarean before labour	Denial of pregnancy	Alone
M05	French (Ivory Coast)	Single	5 years	G1P1- 1st Caesarean	Planned Caesarean before labour	Isolation	Alone
M06	Bafia (Cameroon)	Single	1 month	G1P2-1st Caesarean, twins.	Caesarean during labour	Traumatic experiences	Alone
M07	Bambara (Mali)	Single	1 month	G3P3- 3rd Caesarean, the first 2 in Mali	Emergency Caesarean before labour	–	A female friend

2. Thematic analysis

The thematic analysis detected 4 main themes articulating intimate, inter-subjective and cultural perceptions of Caesarean section with the experience of the care provided. We have named these themes (1) the shock of the Caesarean, (2) isolation and loneliness, (3) the absence of a cultural containing function, and (4) experiences of the medical follow-up.

The verbatims quoted as examples are intended to illustrate the themes and to help the understanding of the reader. The themes are to be considered as reflecting the experience of all the participants, except when the contrary is explicitly stated.

### The shock of the caesarean

The first theme in the results is related to the intimate experience of Caesarean delivery in a setting of surprise, disappointment and pain. Although this particular theme does not apply specifically to migrant women, exemplifying an experience probably common to all women subjected to this experience, its complexity is greater in the context of migration. In almost all the situations here, the participant was unprepared for Caesarean delivery, which was at odds with her idealised projections of childbirth. One participant had the impression of having been deprived of the “magic of childbirth”, while another said she had not had the dreamed-of delivery. Almost all participants said they had hoped for a “natural (or “physiological”) delivery”, or for a “delivery without epidural analgesia”.

MO2 – It was an experience completely different from what I had imagined. I wanted to give birth very naturally, in a natural setting, without epidural or anything else.

MO3 – I wanted a physiological delivery, I had a plan for the birth of the child, I wanted to cut the umbilical cord with delayed clamping.

Only one woman said that she had not formed a childbirth plan, attributing this to lack of time and mental availability to settle down and dream. The context of migration and an atmosphere of health uncertainties relating to Covid-19 hampered maternal reveries.

MO1 - It seems to me that with all the changes in recent years I have in a way got into the habit of not anticipating things […] It's just that our lives have changed so much… moving to France was an enormous life change, a new country, so many challenges.

The announcement and the decision to perform a Caesarean appears as an unwelcome surprise, too sudden, and received with disappointment by the participants who had never envisaged this mode of delivery. The preparation sessions for childbirth were of no help in the situations encountered here in preparing for Caesarean delivery.

MO2 – It was nothing like what I expected it to be. It was very sudden, all the information came too fast, something you are not expecting at all.

P (MO2) It was rather surgical, in fact, rather cold and surgical.

The corollary of this disappointment is the experience of a sort of failure, with feelings of incompetence and inability to bear children, going even as far as the expression of intense guilt. Caesarean delivery in this case is seen as deviant compared to what was systematically referred to as “normal” delivery.

MO5 - I failed…

MO2 - It made me feel like a bad mother, and a bad woman because I couldn't manage it properly - why? Did I do something wrong? Why can't I give birth normally, like other women?

This feeling of failure is stressed by several women, who compare themselves to other women around them, and most often with their own mother. The reference to other women is a constant observation in the interviews, characterising delivery as the life event that places a woman in her lineage and her social group.

MO6 - In my family I am the only one to have had a Caesarean. Otherwise, everyone else has had a vaginal delivery.

The participants were interviewed just after delivery. Their post-operative physical state occupies considerable space in their discourse. Thus fatigue, or even exhaustion, and also pain and worries about recovering from surgery are present in all the interviews. The participants talked of worries about the healing of the scar on the uterus.

MO1 – Everyone looks at the scar, asks me about pain, how things are going. But it's true that we never talk about what's going on inside in any detail. How will it heal?

The procedures encountered in the operating theatre cause distress, fear and anxiety. The lack of privacy on account of the numbers of professionals present, and the accumulation of strong stimuli (noise, lights, milling people) makes the visit to the operating theatre an experience that is a sensory aggression.

MO7 - There they are very quick, there are too many people milling around! There are nurses, lights, bing! bang! You don't know what's going on. It's too much, far too much!

MO4 – When I saw the people with masks, saying "It's alright, don't worry" I was flustered, I was in tears

Caesarean birth is sudden and violent, occurring in an immediacy that overturns psychic temporality of the mother who is thus deprived of the period of labour.

MO2 – We were talking, and the moment we stopped talking they said "there we are, the baby is coming now" and a few seconds later we could hear him cry.

If we look at the words used to describe their Caesarean we note the words “hole”, “big cut” or “they cut your belly open”. Certain participants’ narratives are in very lurid language, or else they give a wealth of details, which suggests that our interviews in the early aftermath had not left time for the experience to be integrated psychically. These raw narratives, without hierarchy in the detail, reflect the violence of the experience, and result from the great psychic vulnerability in which these mothers found themselves at the time of the interviews. They also provide insight into the traumatic potential of Caesarean section. The narratives are characterised by the lexical field of trauma, with the mention of a suspension of time, along with imposed passivity, giving the impression that the woman is placed in the position of undergoing (or enduring) her delivery, no longer an active party.

MO4 – I went all day without eating. There were emergencies, they told me to wait in the other room. And I stayed there waiting till 4 in the morning. It was really hard, I was in pain […] Several times I asked for a drink of water and they said no, I wasn't to eat or drink … so I went more than 24 hours without eating, waiting for my turn.

MO2 – It's as if someone said to you "You are going to die on such and such a day, you have two weeks, 18 days […] You can't compare waiting to die and having a baby, but there…

Caesarean section means an early separation of the mother from her baby, because of the time required to suture the wound and the time in post-operative care. This initial time of separation, lasting several hours, can cause a feeling of abandonment and loneliness for the mother, and the impression of a sudden switch to care of the baby rather than herself. The mother feels alone, abandoned in the recovery room and suddenly no longer the centre of attention of the healthcare team.

MO1 – For me it was the recovery room that was the hardest. I was alone, unfortunately the nurse was not very friendly. I really felt as if I was alone on that floor. There really was no-one around […] I was in a sort of void, it's true. I was no longer the patient, and I was not yet the patient. Because, of course, it was the baby who mattered most […] I felt rather abandoned, in fact, once the baby was taken away.

### Pregnancy and childbirth far from one’s own people: an itinerary characterised by isolation and loneliness

#### Isolation, loneliness and mental distress

The women we interviewed were all suffering intensely from isolation as a result of migration. For those who had maintained contacts with their families, distant family support takes the form of frequent calls and exchanges, mostly several times a day, with the family remaining in the country of origin. When questioned about their experiences of pregnancy the participants all described a pregnancy that was difficult and stressful, with considerable vomiting throughout, the threat of premature birth, or mistaken announcements of miscarriage, and these ordeals were compounded by the pain of separation from the family.

MO3 – In fact it's because I had the risk of a miscarriage. Very early on, at 15 weeks, and then a threatened delivery at 23 weeks. As a result I had to stay in bed, and in fact, as I'm not from here, well my family wasn't there either. So I spent my whole pregnancy alone with my husband.

This feeling of isolation leads to worries about what will happen after the Caesarean and the return home, and this is particularly marked for women with no companion.

MO5 – I just know a few people here and there, but they can't be there for you all the time when you need them. Here everyone has his own occupations, people go to work… You can't call them and ask "can you come and help me".

One of the participants has a history of post-partum depression at the time of an earlier Caesarean delivery, which she links to her loneliness and solitude.

MO7 – When I had my second son I had post partum depression, the real thing! Because overnight, I go to hospital, I have the baby, I come home, and I am alone! I don't even know how to bath the baby, I don't know how to do anything, I was shaking, it was horrible! Horrible – loneliness is … dreadful.

Solitude and distancing from the family as a result of migration were compounded by the closure of borders with the Covid-19 pandemic, and also by lockdown measures. Pregnant migrant women found themselves particularly vulnerable, in a situation of total dependence towards their husbands, who, when present, were their only resource.

MO3 – Suddenly, no longer going to work, not being able to do anything, not being able to get out, I realised that apart from my husband I wasn't going to be able to do anything, or see anyone.

#### The husband, a needed but insufficient support

The husband, when present, has a major role in the pregnancy trajectory of these migrant women. Caesarean delivery imposes considerable requirements on the husband, since the mother in the recovery room is not available for prolonged skin-to-skin with the new-born baby, nor for the first baby care procedures. The two fathers who were present in the interviews (MO1 and M02) were particularly committed fathers, anxious to be present throughout their wife’s time in the maternity unit. All the other participants were isolated and needed to care for the baby on their own. For them, the sadness of being alone, and also the worry of being temporarily incapacitated by the Caesarean and not physically fit to look after the new-born child, were particularly marked.

MO5 – Its the time after the Caesarean [that worries me], because I have practically no-one here. How will I manage? Who will help me?

#### The mother, an omnipresent figure, is cruelly missed

The figure of their own mother appears omnipresent in the discourse of the participants, they miss her cruelly. The participants call on their mothers to accompany them from a distance, to reassure them.

MO1 – My mother is a great person. I called her at one in the morning on three consecutive nights. In the depth of the night she told me "yes, yes I'm with you, it's nine in the morning here! Don't worry". That helped […] just knowing that someone was there. When it's night time you feel so alone […] so it does me good to be able to call my mother"

Several of our participants, especially those from Western African or Sub-Saharan countries express nostalgia for the maternal “portage” they would have had in their own country. The mother of the parturient woman has the role of providing psychological support, physical care and child care, to enable the young woman to regain strength.

MO7 interpreter – Culturally, when the daughter gives birth she will spend one month with her mother, it depends on families, sometimes it's two weeks, sometimes 40 days.

MO5 - So that you can eat well, you are given large portions of dishes at home. There you are given a big fish for you to recover!

MO7 – You eat, you sleep, and you don't see your baby, they look after him.

In addition, the deliveries of their own mothers are systematically recalled by these women. The feeling of having had an abnormal experience is greater among the women whose mothers had vaginal deliveries, while those whose mother underwent Caesarean section identify more easily with that trajectory, which is welcome support.

MO3 - For my mother too her pregnancies were complicated, she had a bad experience of the triggering of labour and Caesarean.

#### Trauma, secrecy, and disgrace

Migrants women can need to conceal their pregnancy from their families back home, which aggravates their isolation and loneliness in confronting these challenges. One of our participants (MO4) was initially in denial of her pregnancy and did not announce it to the family for fear of a negative, violent reaction from her father. For another participant, it was because of the worry of the disgrace she would bring on her family that made her prefer to keep her pregnancy secret.

MO5 - If you aren't married, you can't have a baby… so I preferred not to tell them. As the father and I are no longer together, when things are that way, in the family it's – it's a sort of disgrace in the family. So I don't really want to talk about it, for that reason

For another of our participants (MO6) the pregnancy was the result of rape in the course of migration. The stunned state in which she found herself made the interview distressing, despite the participant’s repeated request not to end it. The interview is punctuated by lengthy silences, and a virtual absence of spontaneous comments to restart the interview by the participant. Her obvious mental slowing, which could be attributed to both the fatigue from the recent Caesarean and to the presence of clear depressive symptoms, can also be seen as a state of dissociation and traumatic numbness.

### The lack of a cultural containing function

#### A negative view of Caesarean section

Four of our participants from Sub-Saharan Africa reported negative cultural representations of Caesarean section. Caesarean delivery is perceived very negatively and there is heavy prejudice for the mother. Caesareans have a bad image first because they inspire fear: in countries with poor medical infrastructures the consequences of surgery – often fatal for earlier generations -are still feared. Caesarean delivery also means having fewer children, thus affecting the woman’s fertility.

MO4 – At that point I was afraid, because at home when we talk about Caesarean delivery, people are scared […] Because it was said that with a Caesarean you can only have three children, that's what they say in Senegal.

Beyond these worries of a medical nature, Caesarean delivery is viewed negatively as an absence of effort, preventing the woman from becoming “a real woman”.

MO7 – When I had the Caesarean, my grandmother said "As for you, you're just lazy, you couldn't even give birth! You had lots of help, didn't you! They open up your belly and they just give you the baby”. For those women it's easy […] Yes, you should have vaginal delivery. That's it, you should… in fact you're not a real woman, not in their heads.

Caesarean delivery belongs to the medical world and makes traditional care impossible.

Caesarean delivery, by essence medicalised, calls into question the place and the presence of the medical profession in childbirth. For women from traditional societies, delivery should be natural and physiological, and excessive medical intervention is dreaded. One participant told about the experience of her own mother, who did not attend the appointment in the maternity unit for labour to be triggered.

MO3 – In African culture, so much medical stuff is not really a good thing. For instance my mother, I know, for one of my brothers, they told her to come in so that could start the labour, and she didn't go, she waited till it started spontaneously.

The women systematically spoke of the traditional care provided for young parturient women having had a vaginal delivery to facilitate their recovery. This care is often described in detail, whether in terms of massages, the intake of certain plants or eating special foods suited to young mothers and favouring recovery. There is however no traditional care in case of Caesarean delivery. Caesareans belong to the world of medicine, and cuts women off from traditional care often provided by the mother or grandmother. This lack of cultural containing echoes with the loneliness and isolation mentioned earlier, which is compounded by the Caesarean delivery which excludes the woman from the traditional care needed for her full integration into the group of women and mothers.

MO6 – I don't know about Caesarean, but with vaginal delivery when it's over you are cared for. Caesarean is a bit complicated. They would touch… it's still complicated. You shouldn't touch the belly, it should be left, you see…

Touch occupies an important place in the care of young parturient women. In intra-cultural situations, a woman who has not migrated and can receive the traditional care thus seems to deliver a second time with the expulsion of the “clots in the belly” (after-birth). Thus the delivery lasts several days, but in this case in the expert hands of women from the family. Everything then suggests that migrant women are dually distanced from their family group, by way of migration first of all, and by the medical profession *via* Caesarean section. The touch of another person, described as so important by the parturient women, is impossible, prevented.

MO5 - When you have a vaginal delivery at home; you can be massaged, someone can massage your belly. It's the mother who does that, or an elderly person, with hot water. They say there are blood clots in the belly and they need to be expelled, so they massage you, they press on your belly, for several days.

The participants who have been able to share things with their family back home all have a particularly strong relationship with their mother, calling her on the phone several times a day and even in the night, and receiving a lot of advice and reassurance. While “our little hints” (MO4) can be used, the woman is nevertheless alone to massage her belly, or to carry out traditional care procedures or prepare special drinks to favour recovery.

MO4 – My mother told me when I wake up in the morning to take a towel or a piece of cloth, dip it in hot water, and then put it on the wound and massage gently, not pressing too hard, but putting a little pressure, and the water should be quite warm even so. She also told me to take millet powder and make it into a porridge, to add palm oil and to drink it hot. That will clean out the belly, wash away the impurities, and all. That will help me have milk in my breasts so I can feed the baby.

#### In a setting of migration, threat to the rituals that welcome a baby

The importance of the rituals for the welcome of a baby are well known. They can take the form of physical, spiritual or symbolic care. The traditional practices intended to instate protection against evil spirits for the mother and her child can be difficult to set up in France. The advice proffered by the mother can contradict the rules imposed by the husband. For one participant in particular (MO5) this violent confrontation concerned traditional and cultural protection measures in contradiction with the religious requirements posed by the husband.

MO4 – I tell my mother that I have done something, but I don't do it, because my husband does not want me to. Because he says to me "That's not written in the Koran, that's traditional stuff, not religious, so…" For him anything that is traditional and not religious, he doesn't do it

In addition, the lack of family and community support and “propping” in France can compromise the traditional rituals for welcoming the child. Among them, the Moslem baptism is practised in the first weeks after birth in Mali which is the country of origin of MO7. Even so, as a migrant mother having only recently arrived in France and with little access so far to other Malians, she does not envisage the baptism “here”. It is a friend, also from Mali and present in the interviews, who acts as a mediator and proposes to introduce the mother and her baby to the local community, asking her own husband to organise the baptism.

Friend of MO7 - In fact baptism within the week is compulsory. It's compulsory, it's religious, it's where you give the baby a first name, when the baby is shaved, they shave his hair. So we'll do it! Now I've seen it, I have my husband, they'll do it, it's compulsory. He'll do it.

### The experience of medical follow-Up

#### Administrative difficulties and difficulties accessing care

Our participants have to cope with numerous administrative difficulties leading to difficulties in accessing care, this being true irrespective of socio-economic position or socio-professional category. Recently-arrived women all report difficulties in finding their way in the French healthcare system, notifying their pregnancy or initiating the monitoring of their pregnancy. They feel lost and in difficulty for access to midwives, gynaecologists and paediatricians in community practice to whom they are generally directed.

MO1 - In fact I have an American friend who moved when she was eight months pregnant or thereabouts. She doesn't speak French, she has no Carte Vitale (personal identification for access to healthcare) of course, and that's difficult… When you are outside the system you are a bit lost.

These difficulties for access to health professionals in ambulatory care continue after childbirth, making the perspective of discharge with no outside support a worrying prospect. One participants expressed her anxiety about the imminent return home, although she had not been able to find a community midwife to visit her at home.

MO3 – When I'm discharged, I need to find a midwife, as I had a Caesarean, a midwife who come to the home. But up to yesterday those that I have contacted are not available.

The language barrier is a considerable difficulty. For one of our participants, also a care professional, it is language that prevents the medical staff from providing medical information to the patients.

MO3 – So they come here, they don't understand the language, nor the explanations. And several times at work I have seen that when the patients don't speak the language well, even the doctor gives fewer explanations because he knows she won't understand. It would be a good thing to always have interpreters in hospital.

#### Antenatal care, preparation for childbirth

There is a stark contrast within the interviews: while most of the participants are very satisfied generally with their stay in the maternity unit after the birth of the baby, the antenatal period is discontinuous and the sessions of preparation for childbirth appear unsuited to their situation. Indeed, all the participants mentioned their difficulty in finding their way in the French health system and finding a community midwife on their own to ensure follow-up of the pregnancy. They repeatedly mentioned not having been able to know who to turn to, having felt alone in organising the follow-up and having experienced considerable anxiety.

MO3 – [In Spain] we have health centres where you find everything, you don't need to look for a midwife. You go there, make an appointment, you say you are pregnant and they organise everything. But here you have to look for a midwife yourself, or a paediatrician or a gynaecologist

Having friends and a network of acquaintances in France facilitates things. Almost counter-intuitively, our most vulnerable participants with regard to the administration and finance, housed in centres for migrants or social accommodation, were accompanied by the social services and were generally better supported in the follow-up of their pregnancy. Psychological care was also systematically offered, showing the considerable concern shown by staff for these difficult itineraries.

MO5 – When I arrived at Port-Royal I was really well supported. I had the support of psychologists, social workers and a midwife. They were really there for me, they answered my questions and they listened to me. They did all they could. It really did me good, it brought me a lot.

Low-risk patients who were oriented towards externalised follow-up in ambulatory care were in contrast less satisfied with their prenatal surveillance. To the difficulties finding their way around the French system and in recruiting community health professionals can be added a feeling of discontinuity in the few appointments in the prenatal period in the maternity unit. When they meet different doctors and midwives at each consultation, the women are unable to share their concerns or worries. This lack of a stable reference is a central element that destabilises the participants, pregnant women and migrants already in a situation of vulnerability. The participants thus clearly expressed their need for caregiving continuity, not just referrals in medical files.

MO2 – Sometimes you see doctors that are completely different, who have your file but that's all. I don't really feel I was properly followed, here, I don't feel really reassured with a person who you can really talk to and all that.

This absence of a frame of reference during pregnancy follow-up relates to a specific mode of organisation in the maternity unit at Port-Royal. We can however add that there are radical differences in care provision linked to differences in protocol between maternity units. One of our participants, who started her follow-up in another Paris maternity unit before transfer to Port-Royal reports a complete change in the mode of healthcare delivery.

MO3 – The problem is that here they don't use at all the same protocol as the one I know. They placed a pessary which is like cervical cerclage, but here they don't use it and they removed it. It was a bit of a shock, because for me the pessary could help! It's a form of support! And I had medication, progesterone and all that, but they said that should go too. So it was a shock, right from the start, because they weren't doing the things I know about. I felt they weren't listening to me.

The sessions of preparation for childbirth appeared inadequate and unsuited to our participants who had ended up with a Caesarean. In all the interviews, we found this impression of sessions that had too little to say about the possibility of Caesarean delivery, and did not enable these women to anticipate this possibility. Sometimes, as for MO2, the preparation sessions were experienced as painful, entailing rivalry with other women, compounding their feelings of “abnormality” associated with Caesarean delivery.

MO2 - I remember one day we were to prepare for childbirth with a community midwife, and we already knew we were programmed for Caesarean – having beside you couples who are saying things, like… "so we feel this and that" when I knew that I wouldn't feel anything at all…

Psychological care was offered to several participants during pregnancy, in the setting of pregnancy denial, depression sometimes compounded by isolation, or a history of trauma. This accompaniment proved to be particularly supportive for certain participants, while others had greater difficulty resorting to it, or regretted having been able to only broach very ordinary subjects in the sessions.

MO3 – I talked with her, we got on well and all that, but she didn't go deep into my feelings. Sometimes I went there, we talked about everything and nothing, but not really about… the fact that I was sad, that I was crying, or things like that…

#### The delivery

The narratives of the participants mingle the difficulties linked to the Caesarean delivery, as seen in the first part of the results, and unanimous recognition of the professionalism and kindliness of the healthcare staff. No participant questioned the decision for Caesarean section despite their negative experience of it, and all agreed to the need for it. Likewise, none of them showed any anger or aggressiveness towards the maternity unit. The narratives are particularly positive and valorising, with the participants expressing their gratitude and satisfaction, stressing the kindliness of the staff and the quality of care in France.

MO7 – They are so nice! I said to myself, these white people are so kind! […] If it was in Africa they would have been saying "Hey, you over there, get out! And who are you? Hurry up!"

However certain elements combine to make the experience of Caesarean delivery distressing. The premises, the organisation of the unit and the status of staff in the unit can be inadequately identified by the parents, compounding feelings of confusion generated by the decision to perform emergency Caesarean section.

Father MO1 – At one moment they moved us to the day hospital, they took us to the pre-labour room or the birth room. There was a notice for a pre-labour room, but they had said birth room.

#### After the birth: the time in the maternity unit

The participants were unanimous in recognising the quality of the care provided by the maternity unit teams, describing the professionals as kind and supportive. The participants, and their husbands when present, expressed their satisfaction and even their gratitude.

MO2 – From the moment we moved to the operating theatre, everyone was really professional, really reassuring and very kind.

MO6 – They coddled me, they took care of me, they tried to see what was wrong. It's reassuring despite everything

The participants had a positive view of the staff visits to their room after the birth by staff they had seen before delivery, doctors, midwives or psychologists, stressing the importance of continuity in the therapeutic and human links with individuals who have been identified. This need for links could be exacerbated in setting of migration where links are often compromised.

MO2 – She [the midwife who announced it] came to see me today, I was glad. To see how things were going

MO5 - I have my psychologist, she came even yesterday, she keeps coming to see me, on Monday, yesterday […] Since the start of pregnancy, it has really supported me. She even told me, if I want, we can stay in contact, even if I see other psychologists out there, I can call her any time even so. She said so.

## Discussion

One hypothesis can be derived from these results. The incision in the Caesarean procedure amplifies and updates the symbolic severance caused by migration. A migrant woman having had Caesarean delivery runs the risk of being excluded from the group of mothers for two reasons. Because of the isolation and loneliness linked to migration, first of all, she cannot be coddled and contained by the female accompaniment she would have had at home (this being independent from the modes and cultural variations of this accompaniment). Above all, it is the painful distancing from her own mother that makes the woman vulnerable, as the transmission of care gestures for parturient women (massages, food to regain strength) and for the care of the infant no longer occurs. These effects, frequently evidenced and well identified as vulnerability factors specific to migrant women in the perinatal period (29–31), are accentuated by the Caesarean. Caesarean section, a purely medical and surgical act, seen at least as having no cultural meaning when it is not viewed as a symbolic or even religious transgression (23), is also deprived of the appropriate care gestures that could provide this act with coherent symbolic and cultural meaning. And on top of that, the young parturient must avoid the traditional care and massages that could favour her recovery.

This study does not claim to be ethnological, nor does it describe how each culture perceives Caesarean section. Indeed, the participants were not interviewed as experts on their culture. The interview guide, while seeking to incorporate cultural elements, focused on the participants’ subjective experiences; the cultural and familial elements that constitute their internalized cultural framework serving as a matrix for their subjective experiences. Thus, the thematic analysis aimed to identify factors of vulnerability linked to the transcultural situation and migration shared by all the participants.

These results casts light on the functions of childbirth and maternity which are specific neither to Caesarean delivery nor to migration. Here everything suggests that the transcultural context aligns with the situation of all women who are becoming mothers. How can one’s integration into the group of adult women be envisaged if one has not been able to prove the ability to give birth? How can one learn to become a mother without the model and support of one’s own mother? Migration, according to Claire Mestre, thus equates to “a quasi-experimental situation that enables us to explore how women need to call on different personal competences and do without the help of their mothers, in particular when they are from a society in which the place of the mother is essential for transmission” (30). Our results underline another important element: the atmosphere of uncertainty in which migrant women find themselves entails the risk of failure to invest fully in the birth of the child, a risk that then involves the child.

The parturient woman’s mother occupies an essential place in the psychological elaboration of pregnancy, with the processes of identification, gratitude and idealisation [Bydlowski, quoted by 30]. The absence of this maternal figure can be partially compensated for by the adoption of a particularly maternal attitude by the husband, as seen in several narratives by our participants. This situation, frequent for migrant mothers distanced from their own mothers, (and more broadly for non-migrants suffering from isolation and loneliness) is accentuated by the physical injury of the Caesarean. The initial prolonged separation from the baby while the mother is in the recovery room, the first care procedures that the woman cannot perform – this makes the father particularly involved in the maternal care of the new-born infant. This is liable to accentuate the gulf with traditional maternal care in the home community and aggravate the mother’s feelings of guilt and incompetence.

Our participants established a parallel between difficult experiences of pregnancy, different from what was expected, and the fact that the pregnancy ended up with a Caesarean. The narratives on these pregnancies show a predominance of the passive register (needing to rest, to stay in bed, vomiting, being restricted in activities, etc.). This passivity is also seen in the experience of Caesarean delivery, where the woman submits to the vicissitudes of protocol and medical decision. The distress relating to intrusion and upheaval linked to pregnancy also entails the necessary experience of passivity that the mother must endure to enable a new being to grow within her body (32). This theme of passivity, inherent in pregnancy and inherent in Caesarean delivery, echoes the situation of passivity to which the migrant woman is subjected, whereby she has to adapt, conform and accept an external cultural framework that differs from her interior cultural framework. Indeed, the logics of healthcare, of life and death entertained by these women may not be in line with the issues and objectives of the medical care provision offered by healthcare teams here (23). This passivity resulting from both migrant status and pregnancy status is reinforced by Caesarean delivery, a painful episode that reinforces dependency towards caregivers and husbands, making the woman unable physically to take care of the baby on her own in the early days. In addition, the importance of pain during labour seems to be missed by certain women who feel they have been forced to be passive as a result of Caesarean delivery, losing control of their own delivery. The violent remarks by traditional grandmothers reported such as “you are just lazy” show the importance of the symbolic dimensions of distress, pain and suffering, seen as legitimising graduation to maternity.

Certain migrant women are particularly exposed to the risk of violence and traumatic events, as with one of our participants whose pregnancy was the result of rape during her travels. Some participants described their deliveries with a wealth of detail involving various sensory modes (visual, auditory or painful scenes) in some cases violent. These details, associated in counter-transference with an impression of diffuse unease, dejection or anger when listening to them, are challenging and of concern to us on account of the ultimate traumatic potential of the event (33, 34). Further to this, the literature outside the migratory setting identifies the risk of traumatic neurosis following a Caesarean delivery (35). We hypothesise that Caesarean delivery could compound the aftermath of the trauma of migration, in other words it could revive and update on the one hand real trauma experienced previously, and on the other the separations and bereavements of the past and the emptiness resulting from migration and distancing from one’s own people. To be validated, this hypothesis requires a longitudinal exploration of a cohort of migrant women having had Caesarean deliveries. We can however also turn to research on trauma in transcultural settings, which can inform us on the modes of activation of experiences of traumatic migration following bodily alterations (36).

One result diverges from the literature and our expectations: none of the women expressed mistrust or contested medical decisions or care protocols. On the contrary, gratitude is unanimous, even when the feelings towards Caesarean delivery are negative. It can be hypothesised that the status of migrant fosters passive social and institutional attitudes (sometimes in extreme cases linked to the inability to understand and to be understood by caregivers). This interiorised passivity could generate narcissistic feelings of frailty and intense dependency towards caregivers and the institution, making expressions of discontent more hazardous.

It can be added that participants may have found it difficult to precisely identify the place of our research interviews in care provision in the institution. The participants indeed mentioned their difficulty in identifying and differentiating the different professionals in the unit, which leads us to think that visits to the patient’s room by a researcher were possibly not sufficiently distinguished from the healthcare staff’s visits, leading to possible confusion.

### Proposals for the improvement of care

Pregnant migrant women need continuity and a healthcare reference in order to develop a feeling of containment throughout the pregnancy, from the start of follow-up until discharge from the maternity facility, and including the operating theatre. It appears fundamental to deploy efforts to reduce moments of isolation and loneliness inflicted on mothers finding themselves alone after their Caesarean, since these moments of isolation reactivate the woman’s feelings of loneliness and abandonment. Likewise, organisational efforts should be deployed to restrict the period of separation between the mother and her baby after a Caesarean. As suggested by Goguikian Ratcliff (37), we also think it is important to search medical files for anamnestic data linked to vulnerability factors among these women, so that they can be taken into account by caregivers: time since arrival in the host country, fluency in the host country language, educational level and urban or rural origin.

All our participants felt insufficiently prepared for a Caesarean. Preparation for a Caesarean should be given more space in sessions preparing for childbirth, so as to reduce the experience of failure and comparisons with other women. Further to this, their own mothers have considerable importance for these migrant mothers. The presence of the patient’s mother should be allowed and encouraged, wherever possible, to foster coherent cultural propping and support and to enable tradition care practices to be maintained. Care should be taken to avoid an ethno-centred stance allowing only the presence of the husband. In the absence of the mother or co-mothers, i.e. other women from the cultural group of origin (11, 38), caregivers, serving as substitute mothers, should endeavour to be “good enough” mothers. They can support motherhood by showing themselves to be sensitive to the cultural identity of these women.

Our results, and the excellent reception of our study by the participants, show that while the initial experience of Caesarean delivery is always difficult, migrant women draw considerable benefit from the opportunity to put words and give meaning to labour and childbirth, which is in favour of a re-appropriation and a return to an active posture after Caesarean delivery that has been experienced passively. We suggest the need to raise awareness among caregivers towards the difficulties of the post-natal period, and the need for systematic debriefing interviews on the birth of the child. In cases of emergency Caesarean, women draw considerable benefit from being able to see the team members present at the time.

In certain maternity units, there are groups of mothers which are open to mothers who have recently given birth. Given the frequency of Caesarean deliveries, it seems relevant to develop groups of women who have had a Caesarean, aiming to enable women to put their feelings of abnormality and guilt into perspective. There are also other systems, such as transcultural consultations, which, using a complementarist approach (Devereux) propose group consultations enabling an affective and cultural “envelope” to be formed for migrant patients with ambivalent representations of the practices encountered here and those of their home country (11, 31, 39).

### Strengths and limitation of the present study

The interviews carried out with the presence of interpreters enabled women whose first language was not French to express their intimate thoughts in their native language. The presence of an interpreter, along with the interview guide specifically constructed to explore cultural perceptions of the subjects’ experiences, were a major methodological concern, favouring the transcultural validity of the research.

The protocol, validated by the Ethics Committee, entails all due precautions to protect the participants in this very vulnerable period. Our objective was not to draw conclusions from experiences specific to one or other culture, but our results do cast light on vulnerabilities exacerbated by the difficulties of migration. Further to this, our exploratory methodology and the very early organisation of the interviews immediately after delivery was not suited to the exploration of phenomena that tend to appear in the later aftermath, such as traumatic neuroses.

A later study could include a larger number of mothers so as to have access to varied modes of care provision, or to explore the mental consequences at a distance from the event. Moreover, recent anthropological studies would be needed to collect cultural representations of C-section by cultural group, and their consequences on femininity and motherhood.

## Conclusion

Caesarean section is a genuine public health challenge, since in France today one in five deliveries is a Caesarean delivery. Migrant women are particularly vulnerable in this respect. Caesarean birth is often experienced negatively and ill-prepared, and it accentuates the distress linked to isolation, feelings of loneliness and the difficulty of becoming a mother without the support of their own mother. The absence of a culturally coherent meaning allocated to Caesarean birth places the migrant women from traditional cultures in a position that devalorises them and causes feelings of guilt. We propose to deploy systematic interviews centred on the experience of Caesarean delivery in the post-natal period, as well as talking groups for women who have had a Caesarean. Migrant women need to be better prepared for this intervention in the course of sessions preparing for childbirth, involving the presence of interpreters. It is urgent to consider migrant women having had a Caesarean as dually vulnerable, and to actively accompany these women for a sufficiently long period before and after the intervention. Maternity unit professionals and mother and child protection centres should be made aware of these problems so that migrant women can develop protective strategies commensurate with their specific vulnerabilities.

## Data availability statement

The original contributions presented in the study are included in the article/supplementary material, further inquiries can be directed to the corresponding author.

## Ethics statement

The studies involving human participants were reviewed and approved by Comité d’Ethique de la Recherche de l’Université de Paris (CER U-Paris). The patients/participants provided their written informed consent to participate in this study. Written informed consent was obtained from the individual(s) for the publication of any potentially identifiable images or data included in this article.

## Author contributions

JR, RR, and MM: conception of the study design, data analysis and interpretation, and final approval of the version to be published. JR: data collection and drafting the article. RR and MM: critical revision of the article. All authors contributed to the article and approved the submitted version.

## Conflict of interest

The authors declare that the research was conducted in the absence of any commercial or financial relationships that could be construed as a potential conflict of interest.

The handling editor BG declared a shared affiliation with the authors at the time of review.

## Publisher’s note

All claims expressed in this article are solely those of the authors and do not necessarily represent those of their affiliated organizations, or those of the publisher, the editors and the reviewers. Any product that may be evaluated in this article, or claim that may be made by its manufacturer, is not guaranteed or endorsed by the publisher.
